# Supplementary material for: Size-Exclusion Chromatography Combined with Ultrafiltration Efficiently Isolates Extracellular Vesicles from Human Blood Samples in Health and Disease
Source: Int J Mol Sci. 2023 Feb 11;24(4):3663. doi: 10.3390/ijms24043663 (PMC9963337; doi:10.3390/ijms24043663)
Supplement: Supplementary file 1 [file ijms-24-03663-s001.zip › ijms-2138833-supplementary.pdf]

SUPPLEMENTARY

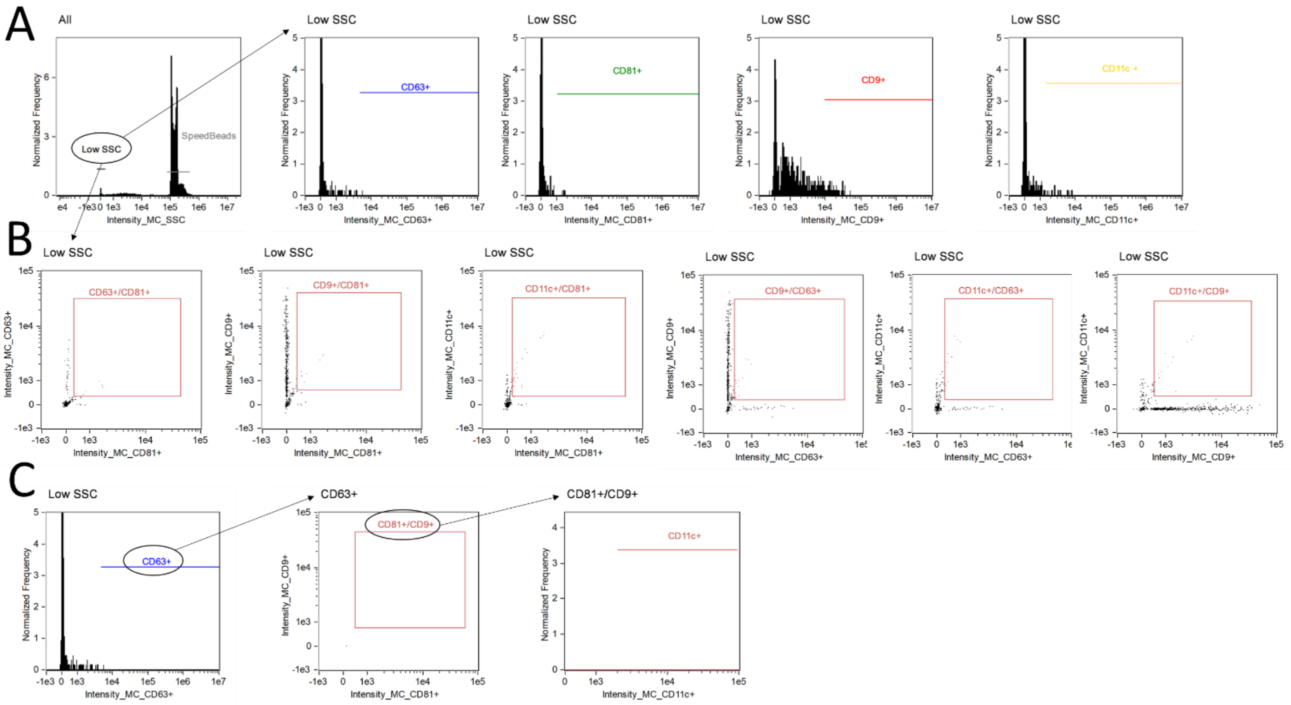

Figure S1. EVs gating strategy applied through imaging flow cytometry. Representative figure of IFC EVs gating strategy by applying the “Intensity_MC” of SSC versus the “Normalized Frequency” in a histogram to identify the Low SSC area limited to the area under the Speed Beads. The graphs show single-positive EVs (A), double-positive EVs (B), and multiple-positive EVs (C) descending from the Low SSC area reporting the fluorescent-positive particles.

Table S1. Protein concentrations in PFP samples and EVs fractions

Protein concentration [mg/mL]			
PFP samples		EVs fractions	
IIM	HDs	IIM	HDs
50.236	52.586	0.068	0.032
52.932	52.642	0.016	0
57.089	49.82	0.025	0
56.688	50.227	0.046	0.044
52.297	52.254	0.103	0.156
57.93	50.909	0.031	0.046
51.544	45.662	0.045	0.044
45.156	52.736	0.105	0.009
47.192	46.975	0.045	0.047
48	50.096	0.092	0.091
52.029	48.151	0.148	0.059
55.647	53.003	0.193	0.113
49.33	64.039	0.048	0.013
51.055	52.382	0.091	0.01
51.648	46.474	0.068	0.057
50.299	48.064	0.065	0.072
52.214	50.454	0.07	0.056
50.081	56.74	0.123	0.085

50.947	48.33	0.088	0.03
52.025	51.782	0.073	0.048
60.307	48.627	0.143	0.171
55.883	50.057	0.081	0.139
55.576	56.817	0.164	0.121
49.932	53.937	0.087	0.025
56.349	57.047	0.32	0.27
62.482	53.018	0.112	0.048
47.227	54.785	0.038	0.154
49.843	46.077	0.056	0.063
49.007	53.371	0.015	0.043
57.039	59.523	0.029	0.189
52.676	52.057	0.041	0.063
52.089	50.68	0.023	0.047
50.371	53.528	0.135	0.256
54.426	53.64	0.047	0.043
53.757	53.9	0.026	0.099
53.464	58.249	0.032	0.024
48.304	53.586	0.031	0.052
60.56	53.669	0.02	0.092
54.08	60.247	0.041	0.127
54.73	51.415	0.067	0.022
48.535	54.219	0.046	0.057
48.409	45.342	0.041	0.029
48.745	55.096	0.135	0.069
62.537	56.812	0.075	0.021
54.547	55.855	0.027	0.064
58.741	52.504	0.032	0.017
56.709	50.124	0.026	0.025
48.131	52.862	0.01	0.063
61.272	51.581	0.166	0
46.472	46.455	0.124	0.045
46.43	57.893	0.027	0.035
53.441	52.254	0.038	0.065
59.366	48.758	0.038	0.093
45.594	41.67	0.038	0.004
44.462	49.592	0.025	0.101
56.276	51.091	0.032	0.043
43.869	53.664	0.048	0.076
49	49.97	0.077	0.041
54.092	49.895	0.054	0.029
56.553	68.84	0.025	0.07
53.018	54.914	0.013	0.244
53.552	49.953	0	0.011
53.702	47.818	0.02	0.032
60.654	49.107	0.038	0.359
49.545	55.683	0.074	0.08

Protein concentration [mg/mL] before SEC isolation (PFP samples) and after SEC isolation (EVs fractions) in both IIM patients (n=65) and HDs (n=65).

Table S2. Mean concentration of positive EVs to the surface markers detected by imaging flow cytometry

	Total EVs samples	Males vs. females	IIM vs. HDs
**CD63+**	$2.65 \times 10^8 \pm 1.97 \times 10^8$	$3.02 \times 10^8 \pm 2.11 \times 10^8$ vs. $2.47 \times 10^8 \pm 1.89 \times 10^8$ ($p = 0.3193$)	$2.91 \times 10^8 \pm 2.07 \times 10^8$ vs. $2.40 \times 10^8 \pm 1.85 \times 10^8$ ($p = 0.3272$)
**CD81+**	$1.53 \times 10^7 \pm 1.89 \times 10^7$	$1.37 \times 10^7 \pm 1.68 \times 10^7$ vs. $1.61 \times 10^7 \pm 2.00 \times 10^7$ ($p = 0.6457$)	$1.62 \times 10^7 \pm 2.06 \times 10^7$ vs. $1.44 \times 10^7 \pm 1.73 \times 10^7$ ($p = 0.7093$)
**CD9+**	$6.17 \times 10^7 \pm 4.77 \times 10^7$	$5.25 \times 10^7 \pm 2.38 \times 10^7$ vs. $6.64 \times 10^7 \pm 5.57 \times 10^7$ ($p = 0.2910$)	$5.89 \times 10^7 \pm 4.78 \times 10^7$ vs. $6.46 \times 10^7 \pm 4.83 \times 10^7$ ($p = 0.6472$)
**CD11c+**	$6.43 \times 10^6 \pm 7.21 \times 10^6$	$6.08 \times 10^6 \pm 6.95 \times 10^6$ vs. $6.60 \times 10^6 \pm 7.42 \times 10^6$ ($p = 0.7952$)	$7 \times 10^6 \pm 7.78 \times 10^6$ vs. $5.85 \times 10^6 \pm 6.68 \times 10^6$ ($p = 0.5413$)
**CD63/CD81+**	$1.89 \times 10^6 \pm 2.70 \times 10^6$	$1.94 \times 10^6 \pm 2.57 \times 10^6$ vs. $1.86 \times 10^6 \pm 2.80 \times 10^6$ ($p = 0.9204$)	$2.14 \times 10^6 \pm 3.06 \times 10^6$ vs. $1.63 \times 10^6 \pm 2.32 \times 10^6$ ($p = 0.4675$)
**CD63/CD9+**	$2.38 \times 10^6 \pm 1.74 \times 10^6$	$2.28 \times 10^6 \pm 1.59 \times 10^6$ vs. $2.43 \times 10^6 \pm 1.82 \times 10^6$ ($p = 0.7459$)	$2.53 \times 10^6 \pm 1.75 \times 10^6$ vs. $2.23 \times 10^6 \pm 1.74 \times 10^6$ ($p = 0.5071$)
**CD81/CD9+**	$4.13 \times 10^5 \pm 6.24 \times 10^5$	$4.98 \times 10^5 \pm 7.71 \times 10^5$ vs. $3.70 \times 10^5 \pm 5.42 \times 10^5$ ($p = 0.4569$)	$4.71 \times 10^5 \pm 6.06 \times 10^5$ vs. $3.54 \times 10^5 \pm 6.47 \times 10^5$ ($p = 0.4711$)
**CD63/CD11c+**	$3.76 \times 10^6 \pm 7.30 \times 10^6$	$4.75 \times 10^6 \pm 9.68 \times 10^6$ vs. $3.27 \times 10^6 \pm 5.85 \times 10^6$ ($p = 0.4632$)	$4.40 \times 10^6 \pm 7.62 \times 10^6$ vs. $3.12 \times 10^6 \pm 7.04 \times 10^6$ ($p = 0.5032$)
**CD81/CD11c+**	$3.28 \times 10^5 \pm 6.22 \times 10^5$	$4.40 \times 10^5 \pm 8.21 \times 10^5$ vs. $2.73 \times 10^5 \pm 4.97 \times 10^5$ ($p = 0.3302$)	$3.33 \times 10^5 \pm 5.50 \times 10^5$ vs. $3.24 \times 10^5 \pm 6.96 \times 10^5$ ($p = 0.9555$)
**CD9/CD11c+**	$2.34 \times 10^6 \pm 4.17 \times 10^6$	$2.85 \times 10^6 \pm 5.30 \times 10^6$ vs. $2.08 \times 10^6 \pm 3.52 \times 10^6$ ($p = 0.5028$)	$2.71 \times 10^6 \pm 4.16 \times 10^6$ vs. $1.96 \times 10^6 \pm 4.21 \times 10^6$ ($p = 0.4934$)
**CD63/CD81/CD9+**	$2.77 \times 10^5 \pm 5.53 \times 10^5$	$3.95 \times 10^5 \pm 7.81 \times 10^5$ vs. $2.21 \times 10^5 \pm 4.05 \times 10^5$ ($p = 0.2630$)	$2.77 \times 10^5 \pm 4.83 \times 10^5$ vs. $2.76 \times 10^5 \pm 6.22 \times 10^5$ ($p = 0.9940$)

Mean concentration [EVs/mL] ± SD of single, double and triple positive EVs to the surface markers in EVs samples (n=60), males (n=20) vs. females (n=40), and IIM patients (n=30) vs. HDs (n=30).

Table S3. EVs concentration and size by nanoparticles tracking analysis measurements

	Total EVs samples	Males vs. females	IIM vs. HDs
**EVs concentration [EVs/mL]**	$1.51 \times 10^{10} \pm 1.06 \times 10^{10}$	$1.46 \times 10^{10} \pm 9.28 \times 10^{9}$ vs. $1.54 \times 10^{10} \pm 1.12 \times 10^{10}$ (p = 0.6737)	$1.71 \times 10^{10} \pm 1.29 \times 10^{10}$ vs. $1.31 \times 10^{10} \pm 7.17 \times 10^{9}$ (p = 0.0306 *)
**EVs mean size (nm)**	201.6 ± 19.04	198.6 ± 19.20 vs. 203.1 ± 18.88 (p = 0.1989)	199 ± 20.02 vs. 204.1 ± 17.78 (p = 0.1227)
**EVs mode size (nm)**	153.4 ± 18.40	151.8 ± 19.04 vs. 154.2 ± 18.11 (p = 0.4718)	151.9 ± 19.81vs. 154.8 ± 16.89 (p = 0.3706)

Mean EVs concentration [EVs/mL] ± SD, mean size (nm ± SD), and mode size (nm ± SD) measured by NTA in total EVs samples analysed (n=130), males (n=44) vs. females (n=86), and IIM patients (n=65) vs. HDs (n=65).

Table S4. EVs concentrations measured by nanoparticles tracking analysis.

**EVs concentration [EVs/mL]**	
**IIM**	**HDs**
1.76×10^{10}	1.43×10^{10}
1.56×10^{10}	1.96×10^{10}
2.47×10^{10}	1.19×10^{10}
1.76×10^{10}	1.6×10^{10}
1.98×10^{10}	1.57×10^{10}
1.08×10^{10}	1.36×10^{10}
1.57×10^{10}	1.71×10^{10}
1.02×10^{10}	1.51×10^{10}
8.5×10^{9}	6.35×10^{9}
1.09×10^{10}	9.46×10^{9}
2.09×10^{10}	1.46×10^{10}
3.45×10^{10}	3.45×10^{10}
1.9×10^{10}	1.99×10^{10}
1.81×10^{10}	1.15×10^{10}
1.26×10^{10}	8.62×10^{9}
2.36×10^{10}	6.95×10^{9}
4.54×10^{10}	3.23×10^{10}
1.88×10^{10}	1.79×10^{10}
3.97×10^{10}	2.17×10^{10}
1.29×10^{10}	2.66×10^{10}
2.08×10^{10}	6.94×10^{9}
6.52×10^{10}	9.99×10^{9}
8.56×10^{10}	3.03×10^{10}
7.59×10^{9}	9.14×10^{9}
1.16×10^{10}	1.13×10^{10}

1.68×10^{10}	8.68×10^{9}
1.3×10^{10}	3.8×10^{10}
1.16×10^{10}	1.34×10^{10}
7.25×10^{9}	1.09×10^{10}
2.03×10^{10}	1.28×10^{10}
1.27×10^{10}	8.9×10^{9}
7.75×10^{9}	6.6×10^{9}
8.31×10^{9}	9.57×10^{9}
1.65×10^{10}	1.1×10^{10}
9.7×10^{9}	1.48×10^{10}
1.95×10^{10}	7.84×10^{9}
2.74×10^{10}	2.61×10^{10}
2.16×10^{10}	1.26×10^{10}
1.39×10^{10}	1.33×10^{10}
1.41×10^{10}	7.15×10^{9}
1.5×10^{10}	5.24×10^{9}
2.03×10^{10}	1.47×10^{10}
1.49×10^{10}	1.34×10^{10}
1.04×10^{10}	6.47×10^{9}
1.06×10^{10}	1.13×10^{10}
1.25×10^{10}	9.04×10^{9}
1.35×10^{10}	1.79×10^{10}
1.08×10^{10}	6×10^{9}
7.6×10^{9}	8.38×10^{9}
4.72×10^{9}	1.4×10^{10}
7.65×10^{9}	9.79×10^{9}
7.09×10^{9}	8.26×10^{9}
1.2×10^{10}	2.2×10^{10}
1.48×10^{10}	4.28×10^{9}
1.13×10^{10}	6.83×10^{9}
7.22×10^{9}	1.2×10^{10}
1.72×10^{10}	6.57×10^{9}
9.99×10^{9}	1.05×10^{10}
1.18×10^{10}	9.82×10^{9}
1.37×10^{10}	9.3×10^{9}
2.5×10^{10}	1.07×10^{10}
8.96×10^{9}	8.63×10^{9}
1.52×10^{10}	5.95×10^{9}
1.83×10^{10}	1.01×10^{10}
7.86×10^{9}	1.11×10^{10}

EVs concentration [EVs/mL] measured by NTA in IIM patients (n=65) and HDs (n=65).
